# Supplementary material for: Learning to quantify uncertainty in off-target activity for CRISPR guide RNAs
Source: Nucleic Acids Res. 2024 Sep 14;52(18):e87. doi: 10.1093/nar/gkae759 (PMC11472043; doi:10.1093/nar/gkae759)
Supplement: gkae759_Supplemental_Files [file gkae759_supplemental_files.zip › SupplementaryDocument.pdf]

Learning to quantify uncertainty in off-target activity for CRISPR guide RNAs
Supplementary Document

Supplementary Figures

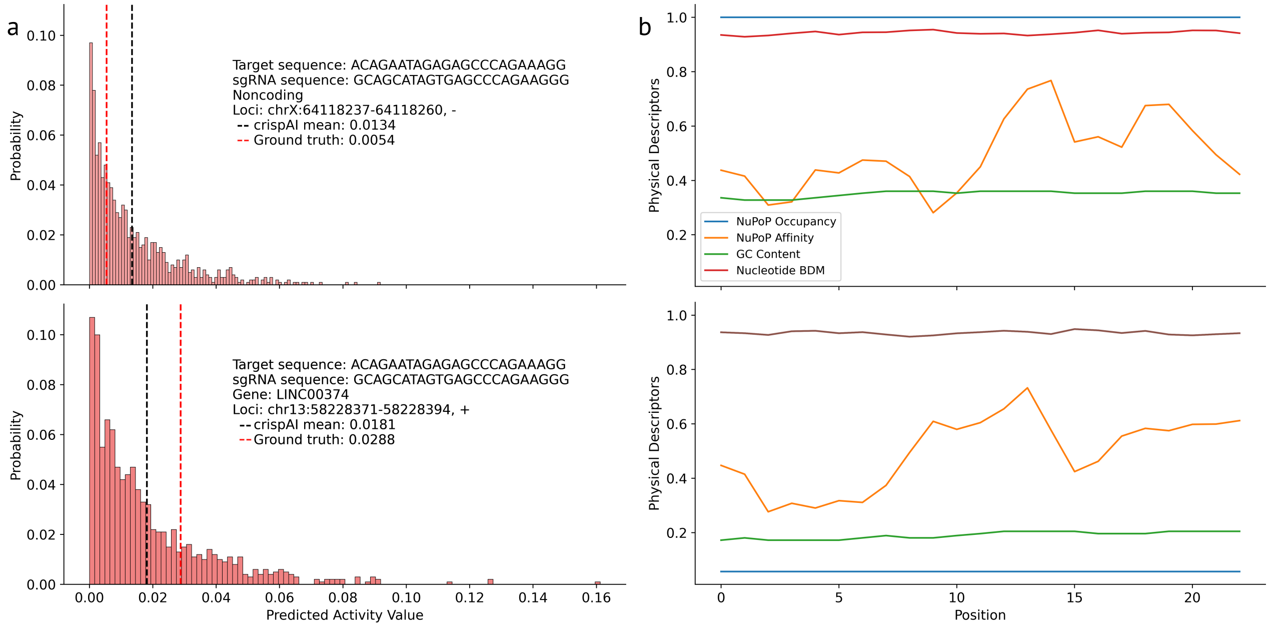

Supplementary Figure 1. We identified regions chrX:64118237-64118260:- and chr13:58228371:58228394:+ on a noncoding region and LINC00374 gene respectively. The regions are present in the test split of CHANGE-Seq dataset where, both sgRNA and target sequences are exactly the same for these two regions. Due to being located on different loci in the genome, their physical descriptors differ. **a.** Figure depicts conditional posterior distributions obtained using crispAI, using the corresponding physical descriptors for both of the samples. The obtained mean crispAI-scores are 0.0134 and 0.0181 and ground-truth detected activity scores are 0.0054 and 0.0288 respectively for non-coding and LINC00374 regions. **b.** The physical features allow the model to differentiate between identical off-target sites through GC content, Nucleotide BDM, NuPoP Affinity and Occupancy scores, within a context window of 147 base pairs.

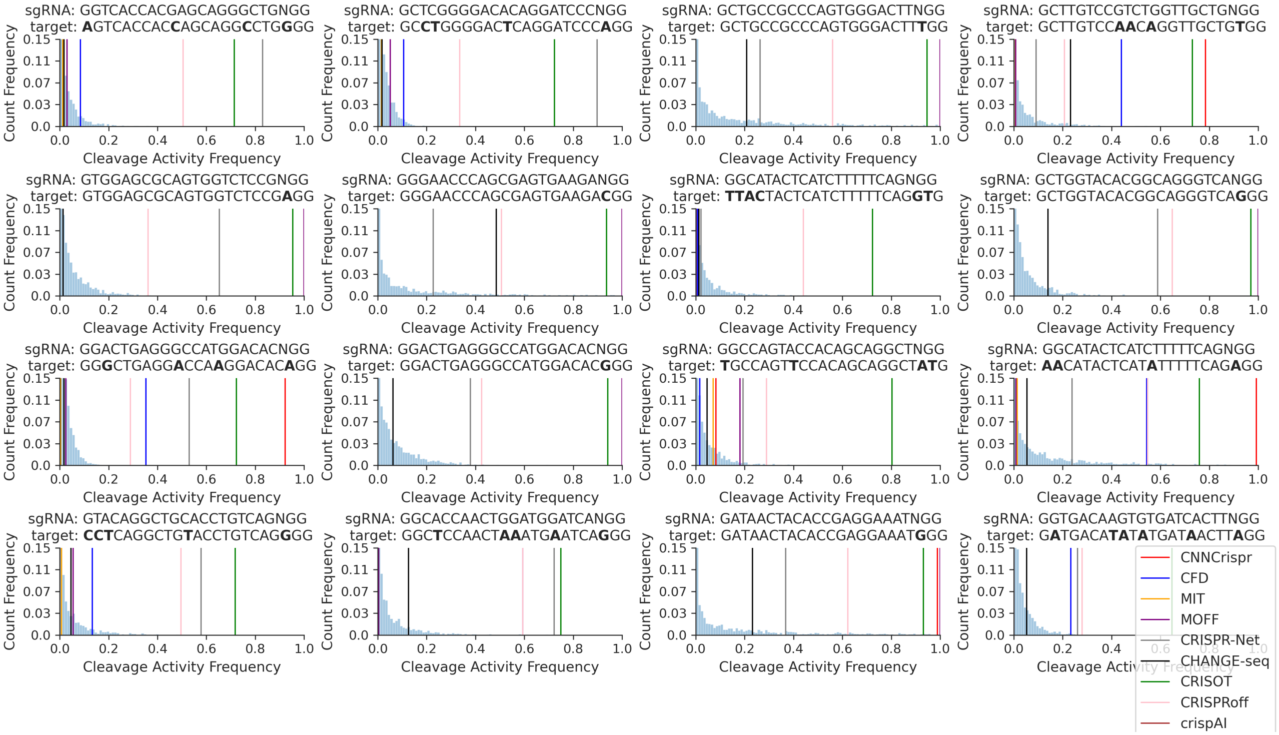

Supplementary Figure 2. Comparison of off-target cleavage activity predictions across different models for 16 randomly sampled sgRNA-target interfaces. The figure shows point predictions from CNNCrispr, CFD-score, MIT-score, MOFF, CRISPR-Net, CRISOT, and CRISPRoff, represented by vertical lines. crispAI predictions are displayed as blue histogram distributions. The green vertical line indicates the ground truth CHANGE-Seq detected frequency. Each subplot presents the sgRNA and target sequences, with mismatches in the target highlighted in bold. The x-axis represents cleavage activity frequency (0 to 1), and the y-axis shows count frequency. This visualization demonstrates the variability in predictions across models, highlighting the heteroscedasticity in off-target activity estimation. crispAI's probabilistic approach provides distributions that capture uncertainty and often align well with ground truth values, especially for low-activity sites where other models show high variability in predictions.

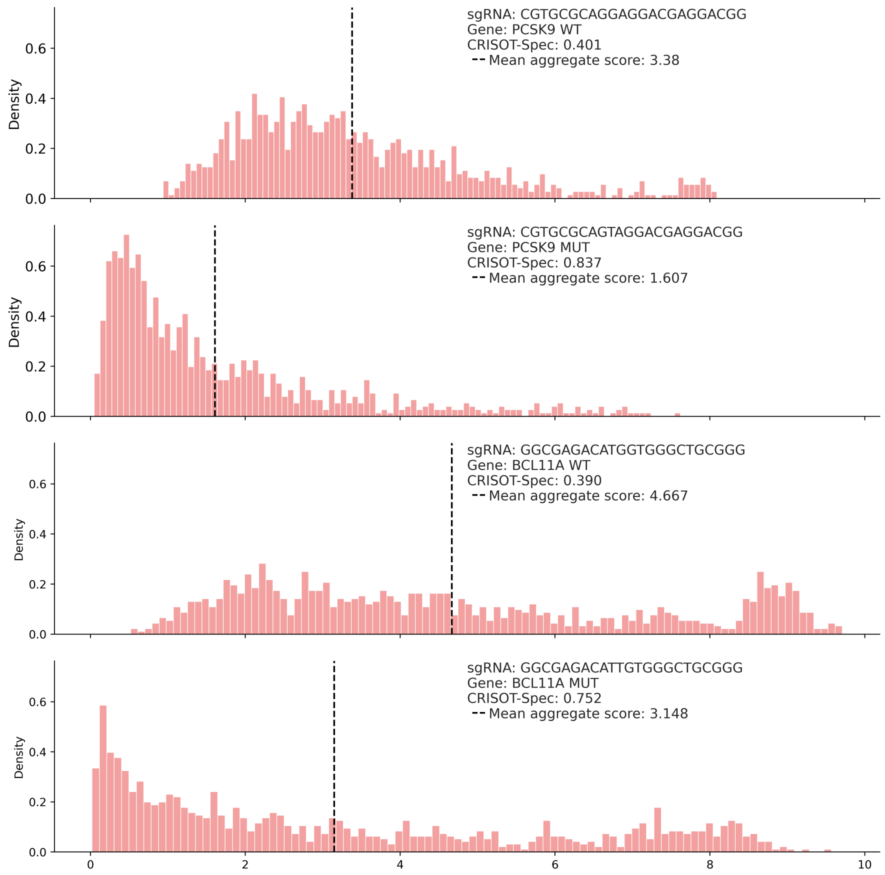

Supplementary Figure 3. We run crispAI-aggregate score on the experimental validation example reported by (Chen et al., 2023). The authors examined two key therapeutic genes, PCSK9 and BCL11A. They reported that CRISPR has been used to reduce cholesterol levels in primates by knocking down the PCSK9 gene. Additionally, they noted that clinical trials are exploring the editing of the BCL11A gene to treat transfusion-dependent β-thalassemia and sickle cell disease. The authors emphasized the importance of ensuring the safety of CRISPR editing on these genes. As a proof of concept, they utilized CRISOT to assess the off-target effects of gene editing and to refine two sgRNAs with inadequate specificities for targeting the PCSK9 and BCL11A genes. We obtained crispAI-aggregate score distributions for both Wild Type and mutated versions of the sgRNA sequences. We observed that crispAI-aggregate scores of the mutated sgRNAs improved 193% and 85%, similarly with the respective CRISOT-Spec scores (108% and 92%). Additionally, we observe that crispAI-aggregate generated posterior genome-wide specificity score distributions are right-skewed for optimized (i.e., mutated) sgRNA sequences compared to wild-type versions. This indicated higher specificity for optimized sgRNA sequences.

Supplementary Tables

– Supplementary Tables 1, 2, 3, 4 and 5 are provided as excel files.

**Model**	**Dataset**					
	**CHANGE-Seq**	**GUIDE-Seq**	**SITE-Seq**	**HEK293T-K562**	**HEK293T**	**K562**
crispAI_NB	0.44762	0.41129	0.21532	0.35635	0.37799	**0.29946**
crispAI_ZIP	0.32512	0.21461	0.08002	0.18748	0.20422	0.11217
crispAI_Poisson	0.00012	0.01582	0.00808	0.03898	0.03165	0.09425
crispAI_ZINB	**0.51142**	**0.45906**	**0.24213**	**0.40695**	**0.46300**	0.28806

Supplementary Table 6. Spearman Correlation coefficients for four predictive models (crispAI NB, crispAI ZIP, crispAI Poisson, and crispAI ZINB) across six different datasets (CHANGE-Seq, GUIDE-Seq, SITE-Seq, HEK293T-K562, HEK293T, and K562). The results reflect the performance of each model in terms of Spearman Correlation coefficients. crispAI ZINB demonstrates higher correlations across all datasets except the K562 cell-line. The models were part of ablation studies comparing candidate zero-inflated and non-hurdle versions of the distributions: Zero-Inflated Poisson (ZIP), Negative Binomial (NB), Poisson, and Zero-Inflated Negative Binomial (ZINB). In these models, the original crispAI model was modified slightly at the output layer to accommodate the chosen distribution and re-trained on the same data with optimized model hyper parameters.

**Distribution**	**Discrete Kolmogorov-Smirnov Test**	
	**Statistic**	**p-value**
Zero-Inflated Poisson (ZIP)	0.2597	0.0
Zero-Inflated Negative Binomial (ZINB)	0.06893	4.578×10^{-296}
Poisson	0.6937	0.0
Negative Binomial (NB)	0.1032	0.0

Supplementary Table 7. Discrete Kolmogorov-Smirnov test results for four distributions: Zero-Inflated Poisson (ZIP), Zero-Inflated Negative Binomial (ZINB), Poisson, and Negative Binomial (NB). The table reports the KS statistic and p-value for each distribution, indicating the goodness-of-fit of the models.

Model	Conv	LSTM	Dense	Spearman Correlation		
				CHANGE-Seq	GUIDE-Seq	SITE-Seq
crispAI_CNN	(256, 3, 1), (64, 1, 1)	N/A	[256, 64, 32], [64, 3]	0.4458	0.3887	0.2704
crispAI_biLSTM	N/A	256	[256, 64, 32], [64, 3]	0.3965	0.3537	0.2476
crispAI_p1	(512, 4, 1), (128, 1, 1)	512	[512, 256, 32], [64, 3]	0.4476	0.4542	0.2467
crispAI_p2	(128, 3, 1), (32, 1, 1)	128	[64, 32, 32], [64, 3]	0.4418	0.4412	**0.2865**
crispAI_p3	(64, 3, 1), (32, 1, 1)	64	[32, 16, 8], [16, 3]	0.4386	**0.4925**	0.2605
crispAI_p4	(1024, 6, 1), (256, 1, 1)	1024	[1024, 512, 64], [128, 3]	0.4416	0.4477	0.2174
crispAI_p5	(256, 3, 1), (64, 1, 1)	256	[256, 64, 32], [64, 3]	**0.5114**	0.4590	0.2421

Supplementary Table 8. Spearman Correlation coefficients for seven predictive models (crispAI_CNN, crispAI_biLSTM, crispAI_p1 to crispAI_p5) across CHANGE-Seq, GUIDE-Seq, and SITE-Seq datasets, along with their architectural configurations. The Conv column details CNN layers, with the first parentheses showing sequence-based features and the second showing physical-based features in the format (filters, kernel size, stride). The LSTM column indicates the hidden size of LSTM layers, with 'N/A' denoting absence. The Dense column presents neuron counts, with the first bracket showing layers for feature processing and the second for output layers from the concatenated representation space. Correlation values range from 0.2174 to 0.5114. crispAI_p5 achieves the highest CHANGE-Seq correlation (0.5114), crispAI_p3 the highest for GUIDE-Seq (0.4925), and crispAI_p2 the highest for SITE-Seq (0.2865). The configurations vary in complexity, with crispAI_p4 having the largest Conv layers (1024, 6, 1), largest LSTM (1024), and most neurons in Dense layers [1024, 512, 64]. crispAI_p3 has the smallest Conv layers (64, 3, 1), smallest LSTM (64), and fewest neurons [32, 16, 8].

Model	Conv	LSTM	Dense	Spearman Correlation		
				HEK293T-K562	HEK293T	K562
crispAI_CNN	(256, 3, 1), (64, 1, 1)	N/A	[256, 64, 32], [64, 3]	0.4087	0.4551	0.2922
crispAI_biLSTM	N/A	256	[256, 64, 32], [64, 3]	0.4216	0.3885	**0.3603**f
crispAI_p1	(512, 4, 1), (128, 1, 1)	512	[512, 256, 32], [64, 3]	0.3742	0.4363	0.2688
crispAI_p2	(128, 3, 1), (32, 1, 1)	128	[64, 32, 32], [64, 3]	**0.4679**	**0.5213**	0.3078
crispAI_p3	(64, 3, 1), (32, 1, 1)	64	[32, 16, 8], [16, 3]	0.4150	0.4944	0.2715
crispAI_p4	(1024, 6, 1), (256, 1, 1)	1024	[1024, 512, 64], [128, 3]	0.4023	0.4316	0.2964
crispAI_p5	(256, 3, 1), (64, 1, 1)	256	[256, 64, 32], [64, 3]	0.4069	0.4630	0.2880

Supplementary Table 9. Spearman Correlation coefficients for the same models in Supplementary Table 8, on the remaining three test sets: HEK293T, K562 and Union.

Model	Spearman Correlation					
	**CHANGE-Seq**	**GUIDE-Seq**	**SITE-Seq**	**HEK293T-K562**	**HEK293T**	**K562**
CFD	0.3331 ± 0.0323	0.3880 ± 0.1262	0.0259 ± 0.0649	0.1889 ± 0.1366	0.2121 ± 0.1751	0.1787 ± 0.2072
MIT	0.3497 ± 0.0482	0.3965 ± 0.1786	0.2287 ± 0.0260	0.3537 ± 0.1447	0.3889 ± 0.1690	**0.2650** ± 0.2521
CNNCrispr	0.0467 ± 0.0525	0.3216 ± 0.2528	**0.2720** ± 0.0419	0.2164 ± 0.1658	0.1915 ± 0.2185	0.1416 ± 0.4884
MOFF	0.4373 ± 0.0401	0.3757 ± 0.2054	0.1263 ± 0.0401	0.1698 ± 0.1291	0.2710 ± 0.1298	0.0910 ± 0.1520
CRISPR-Net	0.4113 ± 0.0373	0.4099 ± 0.1850	-0.1822 ± 0.0704	-0.0032 ± 0.1209	0.1282 ± 0.2860	0.1827 ± 0.2177
CRISOT	0.4494 ± 0.0426	0.4014 ± 0.2043	0.1926 ± 0.0417	0.3334 ± 0.0711	0.4279 ± 0.0799	0.1784 ± 0.1025
CRISPROFF	0.3899 ± 0.0424	0.1799 ± 0.1299	0.0077 ± 0.0552	-0.0073 ± 0.2217	0.0778 ± 0.1114	-0.0181 ± 0.3589
crispAI	**0.5161** ± 0.0393	**0.4509** ± 0.2001	0.2323 ± 0.0431	**0.4194** ± 0.0889	**0.4623** ± 0.0993	0.2629 ± 0.3495

Supplementary Table 10. Spearman Correlation coefficients for different benchmarked models on 10−fold cross-validation setting (CFD, MIT, CNNCrispr, MOFF, CRISPR-Net, CRISOT, CRISPROFF, crispAI) across all test sets: CHANGE-Seq, GUIDE-Seq, SITE-Seq, HEK293T-K562, HEK293T, and K562.

Dataset	Sequence Only crispAI	Physical Descriptors crispAI
**CHANGE-Seq**	0.4498	**0.5114**
**GUIDE-Seq**	0.4403	**0.4591**
**SITE-Seq**	**0.2886**	0.2421
**HEK293T-K562**	0.3441	**0.4069**
**HEK293T**	0.4105	**0.4630**
**K562**	0.2366	**0.2881**

Supplementary Table 11. Comparison of Spearman Correlation coefficients for the Sequence Only crispAI and Physical Descriptors crispAI models across all test datasets. The Physical Descriptors model incorporates GC content, Nucleotide BDM score, NuPoP Affinity, and NuPoP Occupancy scores. This model shows improvements of approximately 14%, 10%, 17%, and 13% over the Sequence Only model on CHANGE-Seq, GUIDE-Seq, HEK293T-K562, and individual cell-lines (HEK293T and K562) datasets respectively. However, a performance decrease of about 14% is observed on the SITE-Seq dataset. Bold values indicate the higher correlation for each dataset.

Model	High Activity	Minimal Activity
CFD	0.1954	0.0455
MIT	0.2634	0.0583
CNNCrispr	0.1787	0.0659
MOFF	0.2168	0.0439
CRISPR-Net	0.2663	0.1029
CRISOT	0.2632	0.0590
CRISPROFF	0.0831	0.0945
crispAI	**0.4529**	**0.1030**

Supplementary Table 12. Spearman Correlation coefficients for CFD, MIT, CNNCrispr, MOFF, CRISPR-Net, CRISOT, CRISPROFF, and crispAI across high activity and minimal activity portions of the CHANGE-seq test dataset. The test set consists of 1,835 off-target sites, divided into minimal activity (11-23 reads) and high activity (23-4844 reads) portions. Correlation values range from 0.0831 to 0.4529 for high activity sites and 0.0439 to 0.1030 for minimal activity sites. crispAI shows the highest correlations in both categories (0.4529 for high activity, 0.1030 for minimal activity). The highest value in each column is highlighted in bold.

Bin	Bin Range	Mean crispAI Reads	Mean Coefficient of Variation	Mean CHANGE-seq Reads	Bin Sample Count & Ratio
**Bin-1**	1.0-5.8	1.5071	1.3932	1.5335	13609 (0.8203)
**Bin-2**	5.8-10.6	7.0753	4.7594	7.4825	1146 (0.0691)
**Bin-3**	10.6-15.4	10.6500	10.0136	12.5669	501 (0.0302)
**Bin-4**	15.4-20.2	11.6625	1.5209	17.8844	294 (0.0177)
**Bin-5**	20.2-25.0	17.0469	6.1345	22.2471	170 (0.0102)
**Bin-6**	25.0-29.8	18.3672	1.2263	26.9508	122 (0.0074)
**Bin-7**	29.8-34.6	22.8969	6.4368	32.2474	97 (0.0058)
**Bin-8**	34.6-39.4	19.0231	1.0547	36.8714	70 (0.0042)
**Bin-9**	39.4-44.2	37.7973	5.1333	41.8906	64 (0.0039)
**Bin-10**	44.2-49.0	41.5590	6.5530	46.3611	36 (0.0022)

Supplementary Table 13. Bin statistics for off-target sites with CHANGE-seq read counts below 50, divided into 10 bins. The table presents bin ranges, mean crispAI predicted reads, mean coefficient of variation for crispAI predictions, mean CHANGE-seq detected reads, and bin sample count with ratio to total samples. Bin-1 (read counts 1.0-5.8) contains 82.03% of samples, while Bin-10 (read counts 44.2-49.0) contains only 0.22%. Mean crispAI reads and mean CHANGE-seq reads are provided for each bin, along with the coefficient of variation indicating prediction variability across bins.

Bin	Bin Range	Mean crispAI Reads	Mean Coefficient of Variation	Mean CHANGE-seq Reads	Bin Sample Count & Ratio
**Bin-11**	50.0-529.4	62.2982	1.6920	128.4875	441 (0.0266)
**Bin-12**	529.4-1008.8	332.2296	2.0018	656.8333	18 (0.0011)
**Bin-13**	1008.8-1488.2	416.0976	2.6390	1264.0	4 (0.0002)
**Bin-14**	1488.2-1967.6	50.8740	3.4987	1590.0	1 (0.0001)
**Bin-15**	1967.6-2447.0	2053.5920	2.0892	2238.3333	3 (0.0002)
**Bin-16**	2447.0-2926.4	1537.1210	1.3881	2768.0	1 (0.0001)
**Bin-17**	2926.4-3405.8	2110.8826	1.9091	3397.0	1 (0.0001)
**Bin-18**	3405.8-3885.2	N/A	N/A	N/A	0
**Bin-19**	3885.2-4364.6	3173.5901	2.2536	4214.0	1 (0.0001)
**Bin-20**	4364.6-4844.0	1748.2245	2.0191	4741.0	1 (0.0001)

Supplementary Table 14. Bin statistics for off-target sites with CHANGE-seq read counts above 50, divided into 10 bins (Bin-11 to Bin-20). The table presents bin ranges, mean crispAI predicted reads, mean coefficient of variation for crispAI predictions, mean CHANGE-seq detected reads, and bin sample count with ratio to total samples. Bin-11 (read counts 50.0-529.4) contains 2.66% of samples, while higher bins contain extremely few samples (0.11% to 0.01%). Bin-18 contains no samples. Mean crispAI reads, mean CHANGE-seq reads, and coefficients of variation are provided for each populated bin, showing the distribution and prediction characteristics for rare, high-activity off-target sites.

Supplementary Notes

Supplementary Note 1. crispAI-aggregate score calculation

Result: crispAI-aggregate score distribution for uncertainty-aware sgRNA genome-wide specificity prediction

$N \longleftarrow$ user-specified number of mismatches (e.g., $N = 5$)
$sgRNA \longleftarrow$ sgRNA sequence of interest
$n_{samp} \longleftarrow$ number of samples to draw from each predicted distribution

offTargets $\longleftarrow$ CasOFFinder($sgRNA, N$) `// search for putative off-target sites`

foreach *offTarget* $\in$ *offTargets* **do**
| posteriorDistributions[offTarget] $\longleftarrow$ crispAI-predicted cleavage activity distribution(offTarget)
end

onTarget $\longleftarrow$ crispAI-predicted cleavage activity distribution($sgRNA$)

for $j \longleftarrow 1$ **to** n_{samp} **do**
| **foreach** *offTarget* $\in$ *offTargets* **do**
| | samples[offTarget][j] $\longleftarrow$ sample from posteriorDistributions[offTarget]
| **end**
| samples[onTarget][j] $\longleftarrow$ sample from onTarget
end

for $j \longleftarrow 1$ **to** n_{samp} **do**
| sumOffTargetSamples[j] $\longleftarrow \sum_{\text{offTarget} \in \text{offTargets}}$ samples[offTarget][j]
| ratio[j] $\longleftarrow \frac{\text{sumOffTargetSamples}[j]}{\text{samples[onTarget]}[j]}$
| crispAI_aggregate_score[j] $\longleftarrow$ log(ratio[j])
end
